# Unlocking the Potential: How Flavonoids Affect Angiogenesis, Oxidative Stress, Inflammation, Proliferation, Invasion, and Alter Receptor Interactions in Endometriosis

**DOI:** 10.1002/fsn3.4607

**Published:** 2024-12-07

**Authors:** Pouya Goleij, Mohanna Khandan, Mohammad Amin Khazeei Tabari, Pantea Majma Sanaye, Dorsa Alijanzadeh, Afsaneh Soltani, Zahra Hosseini, Danaé S. Larsen, Haroon Khan, Alan Prem Kumar, Maria Daglia

**Affiliations:** ^1^ USERN Office Kermanshah University of Medical Sciences Kermanshah Iran; ^2^ Department of Genetics, Faculty of Biology Sana Institute of Higher Education Sari Iran; ^3^ PhytoPharmacology Interest Group (PPIG) Universal Scientific Education and Research, Network (USERN) Tehran Iran; ^4^ Student Research Committee Mazandaran University of Medical Sciences Sari Iran; ^5^ USERN Office Mazandaran University of Medical Sciences Sari Iran; ^6^ School of Pharmacy Zanjan University of Medical Sciences Zanjan Iran; ^7^ Student Research Committee Shahid Beheshti University of Medical Sciences Tehran Iran; ^8^ USERN Office Shahid Beheshti University of Medical Sciences Tehran Iran; ^9^ School of Chemical Sciences The University of Auckland Auckland New Zealand; ^10^ Department of Pharmacy, Faculty of Chemical and Life Sciences Abdul Wali Khan University Mardan Mardan Pakistan; ^11^ Department of Pharmacy Korea University Sejong South Korea; ^12^ Department of Pharmacology, Yong Loo Lin School of Medicine National University of Singapore Singapore Singapore; ^13^ NUS Center for Cancer Research (N2CR), Yong Loo Lin School of Medicine National University of Singapore Singapore Singapore; ^14^ Department of Pharmacy University of Naples “Federico II” Naples Italy; ^15^ International Research Center for Food Nutrition and Safety Jiangsu University Zhenjiang China

**Keywords:** angiogenesis, endometriosis, flavonoids, inflammation, invasion, oxidative stress, proliferation, receptor interactions

## Abstract

Endometriosis, though not classified as a carcinogenic condition, shares features such as oxidative stress, migration, invasion, angiogenesis, and inflammation with tumor cells. This study aims to review the effects of flavonoids on these processes and their molecular mechanisms in preventing and treating endometriosis. A comprehensive review was conducted, involving a literature search in online databases using keywords like “endometriosis,” “endometrioma,” and “flavonoid.” Two authors screened the literature based on predefined criteria, and the selected studies were summarized in a structured data extraction table. Studies reviewed showed that various flavonoids impact key processes in endometriosis, including angiogenesis, inflammation, oxidative stress, and invasiveness. Flavonoids such as 2′,7'‐dichlorodihydrofluorescein diacetate (H2DCF‐DA), naringenin, apigenin, myricetin, 5,7‐dimethoxyflavone (DMF), chrysin, and 6,8‐diprenylorobol were found to induce oxidative stress. Xanthohumol, isoliquiritigenin, and luteolin demonstrated effects on angiogenesis. Apigenin, isoliquiritigenin, and luteolin exhibited anti‐inflammatory properties. Additionally, 3,6‐dihydroxyflavone, isoliquiritigenin, and naringenin displayed anti‐invasive activities. Flavonoid–receptor interactions further enhance their therapeutic potential in endometriosis management. Flavonoids such as nobiletin, chrysin, and daidzein modulate PPARγ and PPARα, reducing inflammation, promoting apoptosis, and improving lipid metabolism. These interactions regulate critical pathways in angiogenesis and immune responses. Additionally, flavonoids impact the aryl hydrocarbon receptor (AhR), with compounds like resveratrol inhibiting cell proliferation and cholesterol biosynthesis, further suppressing lesion growth. The ability of flavonoids like quercetin and kaempferol to antagonize NR4A1 leads to reduced cell proliferation and oxidative stress in endometriotic tissues. These findings offer insights into the mechanisms through which specific flavonoids modulate angiogenesis, inflammation, oxidative stress, and invasiveness in endometriosis. By targeting receptors such as PPARs, AhR, and NR4A1, flavonoids demonstrate the capacity to modulate both metabolic and inflammatory pathways, offering a multifaceted approach to managing endometriosis. Flavonoids can selectively target pathophysiologic molecules and pathways implicated in the condition. Consequently, leveraging the therapeutic attributes of flavonoids could lead to novel strategies for managing endometriosis.

## Graphical abstract

Endometriosis, while not categorized as a carcinogenic condition, exhibits certain notable features such as oxidative stress, migration and invasion, alter receptor interactions, angiogenesis, and inflammation that bear resemblance to molecular aspects of tumor cells. This study aims to examine the impact of flavonoids on oxidative stress, angiogenesis, inflammation, invasiveness, alter receptor interactions, and elucidate their underlying molecular mechanisms in the context of preventing and treating endometriosis.

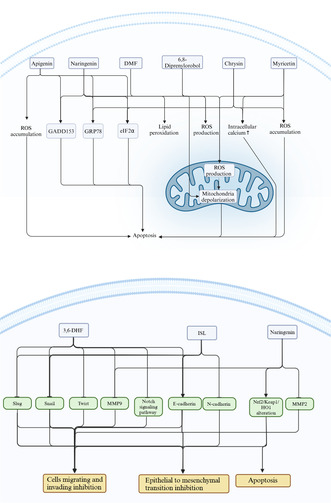

## 1 Introduction

Endometriosis is a common benign chronic condition that primarily affects women of reproductive age. It is characterized by the abnormal growth and proliferation of endometrial cells outside the uterine cavity (Davoodi et al. 2022). Chronic abdominal and pelvic pain, infertility, and dysmenorrhea are frequently reported symptoms associated with endometriosis. Among the affected organs, the ovaries are the most commonly involved in this condition (Vercellini et al. 2014). While endometriosis itself is not considered a carcinogenic condition, it exhibits certain distinctive characteristics, such as proliferation, angiogenesis, migration, and invasion, that bear molecular similarities to those observed in tumor cells (Dall'Acqua et al. 2011; Micozzi and Dog 2004). Several biologic mechanisms are involved in endometriosis pathophysiology, including inflammation (Donnez and Cacciottola 2022), angiogenesis (Rocha, Reis, and Taylor 2013), and oxidative stress (OS) (Scutiero et al. 2017). These can contribute to endometrial cell proliferation and invasiveness (Laganà et al. 2019). The standard treatment approach for endometriosis typically involves oral hormonal therapy. In cases where the disease is severe or does not respond adequately to medical management, surgical intervention is often preferred as a treatment option (Guzick et al. 2011; Hayasaka et al. 2011). Phytochemicals, which are extracted from plants, are essential in preventing and treating diseases, as their bioactive compounds exhibit therapeutic properties like antioxidant, anti‐inflammatory, and anticancer effects (Goleij et al. 2018). Flavonoids are a type of bioactive phytochemicals that can be found in various plants (Elahi et al. 2024). These compounds have been extensively studied, and their therapeutic activities have been demonstrated in several clinical studies including the treatment of infectious diseases (Khazeei Tabari et al. 2021) and chronic illnesses (Khazeei Tabari et al. 2022). Extensive research has been conducted to investigate the preventive activity of flavonoids against both malignant and benign neoplasms (Arefnezhad et al. 2023; Meybodi et al. 2023). The mechanisms of action through which flavonoids exert their effects have been elucidated in previous literature (Mishan et al. 2021). Many different flavonoids were demonstrated to have preventive and therapeutic effects on endometriosis (Gołąbek, Kowalska, and Olejnik 2021). Drawing upon the existing literature, the primary objective of this study is to examine the impact of flavonoids on OS, angiogenesis, inflammation, and invasiveness in the context of endometriosis. Furthermore, this research aims to elucidate the molecular mechanisms through which flavonoids contribute to the prevention and treatment of this condition.

## 2 Materials and Methods

A comprehensive review study was conducted by performing a comprehensive literature search in online electronic databases, including PubMed, Scopus, and Web of Science. The search strategy utilized the following keywords: “endometriosis” OR “endometrioma” AND “flavonoid” OR “flavonoids.” Two authors independently screened the literature based on predefined inclusion and exclusion criteria. The inclusion criteria encompassed original studies that examined the effects of flavonoids on endometriosis. Exclusion criteria involved non‐English articles, review articles, conference papers, unavailable full‐text articles, studies utilizing nonflavonoid phytochemicals, studies employing flavonoid‐rich extracts, and investigations focusing on fractioned extracts (e.g., methanolic and ethanolic extracts). No specific time limitation was imposed on the included studies. Finally, studies that investigated the effects of flavonoids on angiogenesis, inflammation, OS, and invasiveness in endometriosis were selected and summarized in a data extraction table (Table 1).

**TABLE 1:** The effects of flavonoids on different molecular mechanisms in endometriosis.

Flavonoid	Model (cell/animal)	Dose/concentration	Study type	Mechanism	References
3,6‐dihydroxyflavone (3,6‐DHF)	Primary cultured ovarian ectopic endometrial stromal cells (OvESCs) Severe combined immunodeficient (SCID) mice 40 Sprague Dawley rats	0, 5, 10, and 20 μM	In vivo	Anti‐invasive by inhibiting the Notch signaling pathway, increases e‐cadherin Decreasing mRNA expressions of n‐cadherin, twist, snail, and slug Inhibits the migration of OvESCs in a dose‐dependent manner Smaller lesion size in the endometriosis model of SCID mice and Sprague Dawley rats Inhibits the binding of nicd‐csl‐maml complex in OvESCs, thereby inhibiting the expressions of proteins related to notch signaling pathway in vitro Reduced the ectopic lesion size in the in vivo endometriosis model Inhibited the development of EMT in ectopic endometrial stromal cells	(Yu and Zhou 2018)
Chrysin	Human endometriotic End1/E6E7 and VK2/E6E7 cells	0, 5, 10, 20, 50, and 100 μM	In vivo	Apoptotic effects Antiproliferation Induces Ca2+ influx Increases ROS Production Stimulates ER stress by stimulating the unfolded protein response proteins, especially the 78‐kda glucose‐regulated protein–PRKR‐like ER kinase (PERK)–eukaryotic translation initiation factor 2α (eif2α) pathway Regulates the PI3K signaling cascade Inactivated the intracellular phosphoinositide 3‐kinase (PI3K)/protein kinase B (PKB, also known as AKT) signaling pathway in a dose‐dependent manner	(Ryu et al. 2019)
Chrysin	Human epithelial cell lines of endometriosis	0, 10, 20, 30, 50, 80, or 100 μM	In vivo	Antiproliferation Apoptotic cell death (DNA fragmentation elevation) Modulates the expression of the signaling molecules related to cell survival and triggers endoplasmic reticulum (ER) stress in VK2/E6E7 and End1/E6E7 cells Disruption of intracellular homeostasis Induces oxidative stress by ROS Regulation of PI3K/AKT and MAPK signaling pathways	(Park et al. 2020)
6,8‐Diprenylorobol	Human endometriosis‐like cell lines VK2/E6E7 and End1/E6E7	2 μM	In vivo	Apoptotic effects Antiproliferation Induces Ca2+ influx Increases ROS Production Stimulates ER stress Inactivated AKT pathways, activated P38 MAPK pathways Decreased mitochondrial respiration, leading to the reduction in ATP production in VK2/E6E7 and End1/E6E7 cells Induces loss of MMP Downregulates the phosphorylation of the intracellular signaling pathway	(Song et al. 2022)
Apigenin	Endometrial stromal cell line	10, 50, 100 mM	In vivo	Suppresses TNF‐a Antiproliferation Anti‐inflammatory	(Suou et al. 2011)
Apigenin	Human endometriosis cell lines (VK2/E6E7 and End1/E6E7)	0, 5, 10, 20 μM	In vitro	Reduce proliferation Induce cell cycle arrest and apoptosis via the ERK1/2, JNK, and AKT cell signaling pathways Disrupts mitochondrial membrane (mitochondria‐dependent apoptotic pathways) Increases calcium ion concentration of in the cytosol and pro‐apoptotic proteins including Bax and cytochrome c Increases ROS, lipid peroxidation, and ER stress Activates unfolded protein response (UPR) regulatory proteins	(S. Park et al. 2018)
Kaempferol Naringenin Apigenin	Human endometrial stromal cell line		In vivo	Induced decidualization genes, PRL and CNR1, in HESC human endometrial stromal cells Dose‐dependently induced PRE‐luciferase in human endometrial stromal cells (HESC) that is antagonized by RU486	(Toh et al. 2012)
Luteolin Kaempferol	Human endometriotic 12Z cells	15, 30, and 60 μM	In vivo	Apoptotic effects by activating caspase‐3, −8, and −9 Downregulates the expression of the chemokines CCL2 and CCL5 required for monocyte/macrophage influx at endometriotic sites Downregulates the expression of M2 phenotype markers and endometriosis‐promoting factors in macrophages stimulated by human endometriotic cells	(Woo, Jang, and Choi 2021)
Myricetin	20 Female C57BL/6 mice (8 weeks old) Human endometriosis cell lines (VK2/E6E7 and End1/E6E7)	30 mg/kg 0, 5, 10, 20, 50, and 100 μM	In vivo In vitro	In vitro: hampers cell growth and induces apoptosis Disrupts the homeostasis of intracellular organelles by ROS generation and p38 activation and phosphorylation without caspase activation Activation of MAPK and PI3K/AKT intracellular signaling pathway, phosphorylation of ERK1/2 and p70S6K Inhibits the mRNA expression of CCNE1, regulating the development and growth of endometriosis by cell cycle arrest In vivo: Decreases the volume of lesion with decrease Ccne1 expression	(Park, Song, and Lim 2020)
Naringenin	Adult female SD rats (180–200 g) Endometrial stromal cells	50 mg/kg bwt/day 0.1 μM, 0.25 μM, 0.5 μM, 1 μM, 2 μM, 5 μM and 10 μM	In vivo In vitro	In vivo*:* Reduced endometrial lesion growth, volumes and weight Reduced size and number of glands decrease serum TNF‐α and NO level endometriotic lesions In vitro*:* Anti‐proliferative effect Induces apoptosis, ROS production in mitochondrial membrane damage Restore Bcl‐2, caspase‐3, Cyt‐c, and PCNA Reduces the expression of TAK1, PAK1, and VEGF Inhibits Nrf2 expression restored the expression of HO1, NQO1, and Keap1 Anti‐metastatic properties: reduces number of cells migrating by reducing MMP‐2 and MMP‐9 expression	(Kapoor et al. 2019)
Naringenin	Endometriosis cell lines (vaginal mucosa derived VK2/E6E7 and endocervix epithelial derived End1/E6E7)	0, 20, 50, and 100 μM	In vitro	Suppresses proliferation and increases apoptosis by: depolarization of mitochondrial membrane potential pro‐apoptotic proteins Bax and Bak Increases ROS, endoplasmic reticulum (ER) stress through activation of eIF2α and IRE1α, GADD153, and GRP78 proteins Activation of MAPK and inactivation of PI3K pathways	(Park et al. 2017)
Xanthohumol	Mice 10 to 14‐week‐old female BALB/c mice	100 mM	In vivo	Decreases the size of the lesions Reduces level of phosphoinositide 3‐kinase protein Anti‐vascularization Does not induce apoptotic cell death of endothelial cells	(Rudzitis‐Auth et al. 2012)

## 3 Effects of Flavonoids on Oxidative Stress in Endometriosis

OS refers to an imbalance between prooxidants and antioxidants, characterized by an excessive presence of prooxidant agents (Khazeei Tabari et al. 2021). Endometrial cells, like other cell types, can be adversely affected by OS, leading to disturbances in the activity of antioxidant and prooxidant enzymes. Research has demonstrated that patients with endometriosis exhibit reduced antioxidant system activity compared to their healthy counterparts (Prieto et al. 2012). The proliferation and damage found in endometriotic cells are noteworthy effects of OS on the disease pathophysiology (Samimi et al. 2019).

There is widespread recognition that various biomarkers and enzymes are upregulated in response to elevated cellular OS. Catalase, a well‐known enzyme involved in cellular protection against OS, has been found to be increased in endometriotic cells compared to those of healthy women. This observation suggests a potential correlation between OS and the development of endometriosis (Turkyilmaz et al. 2016). In addition, OS has the capability to upregulate the mitogen‐activated protein kinase (MAPK)/extracellular signal‐regulated kinase (ERK) pathway. Activation of this pathway has been implicated in promoting the proliferation of endometriotic lesions and enhancing cell survival (Tosti et al. 2015). The presence of iron overload in patients with endometriosis has been associated with elevated levels of reactive oxygen species (ROS). This increase in ROS can subsequently lead to the activation of NF‐κB in macrophages present in the peritoneum (Defrère et al. 2008). ROS can damage cells by impairing lipid peroxidation, oxidating proteins, and inducing DNA breaks (Juan et al. 2021). Several lines of evidence suggested the increase of ROS in endometriotic cells as a potential mechanism for damaging endometriotic cells and anti‐endometriosis effects of flavonoids.

Park and colleagues (Park et al. 2017) treated human endometriosis cell lines (VK2/E6E7 and End1/E6E7) with 10 μM 2′,7′‐dichlorodihydrofluorescein diacetate (H2DCF‐DA). The cells were washed with PBS twice and then treated with naringenin (0, 20, 50, and 100 μM). The H2DCF‐DA was oxidized when peroxides were present and changed to 2′,7′‐dichlorofluorescein (DCF), a fluorescent substance used to assess the production of intracellular ROS by flow cytometry. The findings showed that intracellular ROS could be increased in cells treated with naringenin (20–100 μM) dose‐dependently. The greatest effect was noticed at 100 μM, which increased ROS production three and two times in VK2/E6E7 and End1/E6E7 cell lines, respectively. The activation of eIF2α and several other proteins, including glucose‐regulated protein 78 (GRP78) and growth arrest and DNA damage‐inducible gene 153 (GADD153), were slightly higher in the treated cells compared to the control group, which suggests that activating these proteins could be a possible mechanism in ROS‐induced cell death caused by naringenin. Interestingly, at lower doses (0.5, 1, and 5 μM), naringenin could also induce cellular and mitochondrial ROS in endometrial cells dose‐dependently, and at 5 μM, naringenin depolarized the mitochondria and had the greatest effect on apoptosis (Kapoor et al. 2019).

Apigenin (4′,5,6‐trihydroxyflavone) can be produced through the phenylpropanoid pathway and can be obtained from phenylalanine and tyrosine (Salehi et al. 2019). In another study on human endometriosis cell lines (VK2/E6E7 and End1/E6E7), apigenin (5, 10, and 20 μM) could lead to ROS accumulation. Furthermore, lipid peroxidation was upregulated in both cell lines. GADD153, GRP78, and eIF2α were also found to be increased in cells treated with apigenin. Myricetin (20, 50, and 100 μM) could also exert the same effects as the apigenin and naringenin on OS. The mitochondrial depolarization following the increased level of ROS was associated with cell death in VK2/E6E7 and End1/E6E7 cell lines. At the highest concentration (100 μM), myricetin increased the calcium and intracellular ROS accumulation (Park, Song, and Lim 2020).

In a study, it was demonstrated that 5,7‐dimethoxyflavone (DMF), a flavonoid derived from *Kaempferia parviflora*, effectively increased levels of reactive oxygen species (ROS) and lipid peroxidation. The study used concentrations of DMF at 20, 50, or 100 μM. Interestingly, the concentration at which the highest ROS production occurred varied between human endometriosis cell lines, specifically VK2/E6E7 and End1/E6E7. The authors reported that the highest induction of ROS was observed at DMF concentrations of 50 μM for End1/E6E7 and 100 μM for VK2/E6E7. Furthermore, the study suggested that the increase in ROS levels triggered endoplasmic reticulum stress and subsequently affected the expression of MAPK proteins, p53, and JNK (Park et al. 2020).

**FIGURE 1:**
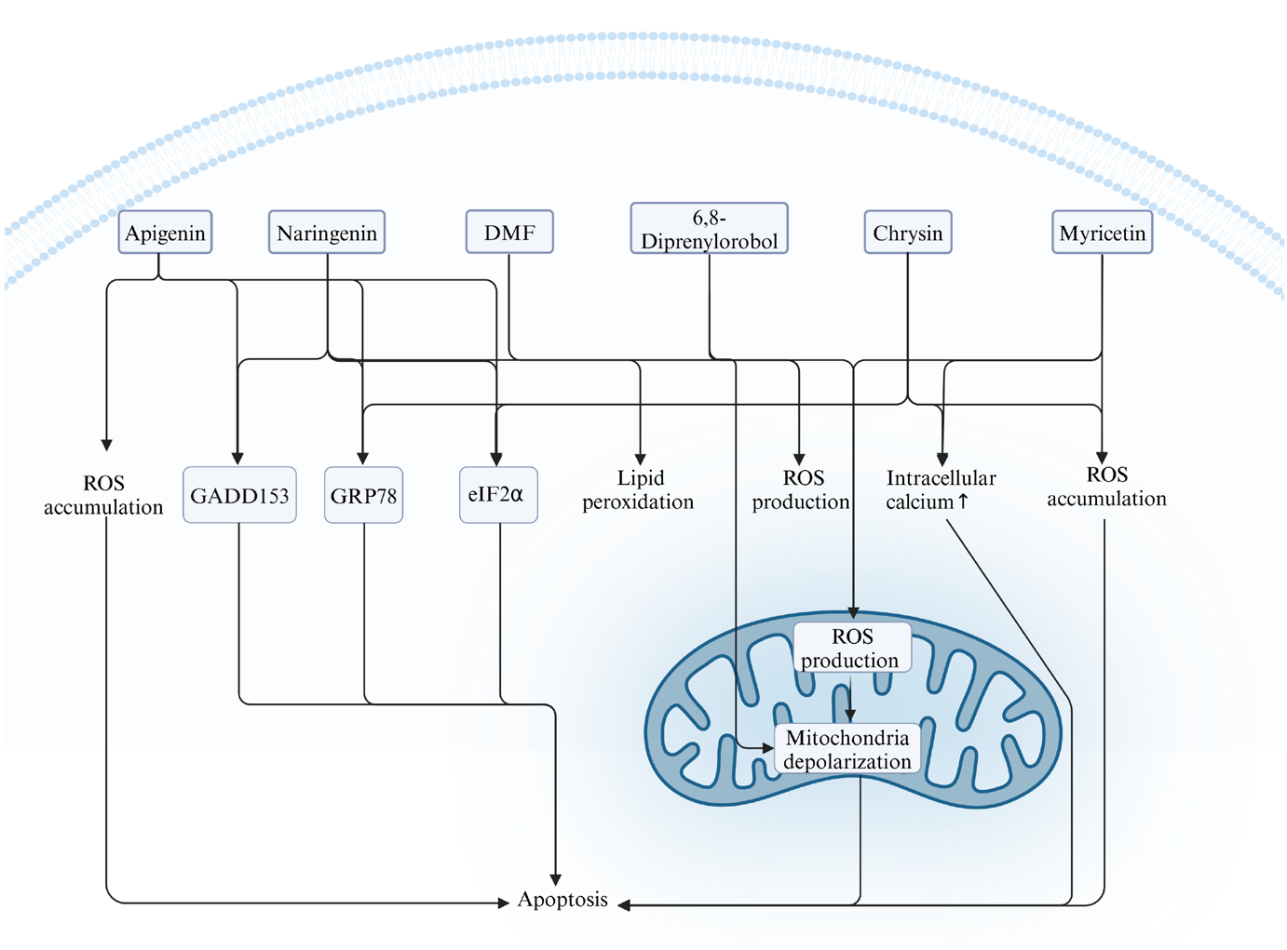
Apigenin, naringenin, DMF, 6,8‐diprenylorobol, chrysin, and myricetin can modulate different mediators related to the OS in the endometriosis cells, leading to increased ROS production in mitochondria and cytosol, which can lead to apoptosis. Created with BioRender.com.

Chrysin, also known as 5,7‐dihydroxyflavone, is a flavone compound consisting of 15 carbons. In a study, it was observed that chrysin, when administered at doses of 5, 10, and 20 μM, significantly increased the levels of calcium and reactive oxygen species (ROS) in VK2/E6E7 and End1/E6E7 cells. Consequently, the authors concluded that the apoptosis induced by chrysin treatment in the cells was mediated by the production of ROS and the elevation of calcium levels in the cytoplasm. Consistent with previous studies, the study also found upregulation of GRP78 and eIF2α, further supporting the role of flavonoids in increasing OS by triggering GRP78 and eIF2α in cells (Ryu et al. 2019). In a study conducted by Song et al., another flavonoid called 6,8‐diprenylorobol, extracted from *Cudrania tricuspidata* , was investigated for its potential inhibitory effects on endometriotic cell lines. Different concentrations of this flavonoid, namely 0.1, 0.2, 0.5, 1, and 2 μM, were utilized. The intracellular ROS assay, employing 2′,7′‐dichlorofluorescein diacetate (DCFH‐DA), revealed that at a concentration of 2 μM, 6,8‐diprenylorobol induced the highest increase in ROS production in both VK2/E6E7 and End1/E6E7 cells. Additionally, the substance was found to depolarize the mitochondrial membrane. 6,8‐Diprenylorobol, similar to previously mentioned flavonoids, can induce mitochondrial dysfunction, leading to cell damage by increasing cellular ROS levels (Song et al. 2022). Based on the studies discussed, it can be inferred that one mechanism through which flavonoids exert their anti‐endometriosis effects is by increasing intracellular ROS and calcium levels. This elevation of ROS and calcium induces OS within the endometriotic cells, ultimately leading to cell death. Thus, the ability of flavonoids to promote OS through ROS and calcium modulation contributes to their anti‐endometriosis effects. Another study on endometriosis mice showed an increase in superoxide dismutase (SOD) levels and a decrease in vascular endothelial growth factor (VEGF) levels in endometriosis mice by a flavonoid extracted from *Phaleria macrocarpa*, therefore showing proper antioxidant effects (Sutrisno et al. 2023; Figure 1).

## 4 Effects of Flavonoids on Angiogenesis in Endometriosis

Angiogenesis plays a critical role in the development of endometriotic lesions and the ectopic implantation of endometrial tissue. This complex process requires the interaction of multiple molecules. Among these molecules, VEGF is considered one of the most crucial factors involved in regulating angiogenesis in endometriosis (Dull et al. 2019). The presence of blood vessels within endometriotic lesions is essential for the development and progression of endometriosis. Angiogenesis, the formation of new blood vessels, is regulated by various mediators, including VEGF, tumor necrosis factor‐α (TNF‐α), and IL‐6 (Khazeei Tabari et al. 2022). Studies have demonstrated that patients with endometriosis exhibit significantly higher concentrations of these mediators in their peritoneal fluid. This suggests that the dysregulated expression of VEGF, TNF‐α, and IL‐6 contributes to the abnormal angiogenic processes observed in endometriosis (Ono et al. 2021).

A prenylated flavonoid derived from hops known as xanthohumol is known to have pleiotropic antineoplasm effects by influencing various cellular processes. For the first time, Rudzitis‐Auth et al. (Rudzitis‐Auth et al. 2012) showed that xanthohumol prevents endometriotic lesions from growing and becoming vascularized. The anti‐angiogenic properties of xanthohumol were discovered in this study. Xanthohumol at a concentration of 100 mM and a dosage of 8 mg/kg body weight was found to effectively suppress angiogenesis, resulting in a noticeable reduction in vascularization of endometriotic lesions. In the control group, uterine grafts exhibited typical endometriotic lesions characterized by dilated glands resembling cysts, surrounded by a vascularized endometrial stroma. However, in contrast to the control group, xanthohumol treatment significantly reduced the size of these lesions on Day 28, regardless of their location within the peritoneal cavity. This reduction in lesion size was accompanied by a decrease in phosphoinositide 3‐kinase protein levels. Furthermore, the suppression of vascularization in xanthohumol‐treated lesions was evident by a significantly lower density of microvessels per square centimeter on Day 28 compared to the control group, which received vehicle treatment. Multiple mechanisms are likely involved in mediating the anti‐angiogenic effect of xanthohumol. The study also demonstrated that xanthohumol inhibits microvessel proliferation in endometriotic lesions without inducing endothelial cell death, possibly due to its direct inhibition of VEGF signaling.

Isoliquiritigenin (ISL), a naturally occurring flavonoid, has been identified in the roots of shallot ( *Allium cepa* ) and licorice (*Glycyrrhiza uralensis*). Previous studies have shown that ISL exhibits antitumor properties. Hsu et al. (Hsu et al. 2020) studied a design to find the effects of isoliquiritigenin (ISL) on endometriosis in vivo. ISL (1 and 5 mg/kg for 4 weeks) inhibited angiogenesis by reducing the concentration of VEGF and endometriotic lesions in mice.

Basil ( *Ocimum basilicum* L) leaves include different flavonoids such as nevadensin, rutin, salvigenin, cirsileol, eupatorin, quercetin, kaemferol, cirsimaritin, and glycosides. In an in vitro study, endometriosis mouse models received a dose of basil leaf ethanol extract in control and study groups. The result of this study showed a decrease in VEGF expression, also limiting the severity of endometriosis lesions (A'yuni, Sa'adi, and Widjiati 2023). Another study in the peritoneal tissue of endometriosis mice models in both in vitro and in vivo study showed that genistein, another flavonoid, could reduce angiogenesis and ER‐*α* expression causing the decrease in proinflammatory and inflammatory cytokines (Sutrisno and Maharani 2024).

In an in vitro study, endometrial cancer cells were incubated with different doses of vitexin. The result showed that vitexin as a flavonoid can reduce angiogenesis in endometriosis cell line (Liang, Jiang, and Sun 2023).

M2 macrophages express proangiogenic substances, including VEGF, with higher VEGF levels found in the peritoneal fluid of endometriosis‐affected women. Activated peritoneal fluid macrophages produce and secrete VEGF, and VEGF produced from macrophages promotes angiogenesis in endometriosis‐affected women. Collectively, results point to alternate stimulation and inhibition of macrophage recruitment as viable therapeutic approaches for endometriosis (Hogg, Horne, and Greaves 2020). Woo, Jang, and Choi (2021) found that luteolin (15, 30, and 60 M) reduced monocyte/macrophage infiltration through CCL2 and CCL5 at endometriotic locations. So, as a result, luteolin could inhibit VEGF produced by macrophages.

## 5 Effects of Flavonoids on Inflammation in Endometriosis

Flavonoids have demonstrated significant anti‐inflammatory effects in a range of diseases (Abdollahi et al. 2023). Estrogen stimulation of intraperitoneal macrophages has been identified as a causative factor for inflammation and the growth of endometriosis. This stimulation promotes the production of TNF‐α and IL‐1 in endometriotic cells, thereby activating TNF‐β and increasing the release of IL‐6 and IL‐8 (Hendri 2021). The pathophysiology of endometriosis is believed to involve a localized inflammatory response within the peritoneal environment. Based on different review articles, flavonoids can show anti‐inflammatory properties (Tassinari et al. 2023).

A study on LPS‐induced endometrial carcinoma Ishikawa cells showed the anti‐inflammatory properties of safflower's flavonoids by regulating the PI3K/AKT signal pathway to treat the inflammatory injury of Ishikawa cells (Chen et al. 2023). Flavonoid of *Phaleria macrocarpa* reduced IL‐17A levels in endometriosis model mice and decreased inflammatory factors (Prastiwi et al. 2023).

A study conducted on women with endometriosis found elevated levels of the inflammatory proteins interleukin IL‐6, IL‐8, and TNF‐α in their peritoneal fluid, indicating their potential contribution to the inflammatory processes associated with the condition (Iwabe et al. 1998). They also demonstrated that TNF‐α and IL‐8 increase mitogenic activity and that TNF‐α upregulates IL‐8 expression in endometriotic stromal cells (ESCs) by activating nuclear factor (NF‐kB) (Arlier, Kayisli, and Arici 2018). Women with endometriosis have elevated prostaglandin E2 (PGE2) levels in their peritoneal fluid (Yi et al. 2020). Cyclooxygenase (COX)‐2 can control the survival and invasion of endometriotic cells, and it is more frequently expressed in ectopic endometria than in eutopic endometria (Banu et al. 2008).

In a research by Suou et al. (2011), it was demonstrated that apigenin (10, 50, and100 μM) can modulate the NF‐ kB pathway in endometriotic stromal cells, which inhibited TNF‐α‐ induced cell proliferation and prostaglandin E2 expression. Apigenin was found to reduce mitogenic activity and inflammatory response in endometriotic stromal cells. Based on these findings, apigenin emerges as a promising therapeutic option for managing inflammation in endometriosis. In a study by Hsu et al. (Hsu et al. 2020), female Balb/c mice were given ISL (1 and 5 mg/kg) or vehicle for 4 weeks after being surgically induced to have endometriosis by transplanting uterine tissue into the abdominal cavity. The administration of ISL therapy resulted in a reduction in both the size and weight of endometriotic lesions. This therapy was also associated with a decrease in IL‐1 and IL‐6 levels in the bloodstream, as well as a reduction in inflammatory cytokines within the lesions. Additionally, microscopic examination revealed that ISL therapy inhibited epithelial to mesenchymal transition (EMT) and induced apoptosis in the lesions.

Park et al. (2024) conducted both in vitro and in vivo experiments on transplant mouse model and patient‐derived immortalized human ovarian endometriotic stromal cells respectively with the administration of Baicalein. The results of this study showed that this flavonoid can reduce endometriosis progression and also the expression of proinflammatory cytokines in endometriotic lesions and endometriosis cell line. These effects can occur through the increase of mitochondrial calcium flux and ROS generation.

Another study showed that vanillin (a flavonoid) significantly suppressed the growth of endometrial lesions and decreased the NF‐κB signaling pathway, pro‐inflammatory cytokines, and ROS compared to control group (Liu et al. 2023).

In a study by Canday et al. (2024), female rats were used as an endometriosis model. This study focused on TNF‐α, VEGF, interleukin 6, interleukin 8, myeloperoxidase, catalase, and anti‐mullerian hormone values, MMP‐1, nitric oxide, superoxide dismutase.

Studies have demonstrated that endometriotic tissues in endometriosis exhibit increased production of chemokines, including C‐C motif chemokine ligand 2 (CCL2) and CCL5. This augmented chemokine production fosters a pro‐inflammatory microenvironment, which contributes to the progression of the disease by facilitating the recruitment of macrophages to endometriotic lesions (Anupa et al. 2020). According to research by Woo, Jang, and Choi (2021), luteolin (15, 30, and 60 M) induces apoptosis in human endometriotic cells. It inhibits the activation of endometriosis‐associated macrophages (EAMs), suggesting that luteolin's anti‐inflammatory properties are linked to its inhibitory effect on EAMs and endometriotic cells.

## 6 Effects of Flavonoids on Proliferation and Invasion in Endometriosis

The initial stage in the formation of an endometriotic lesion involves the adherence of endometriotic cells to the layer of mesothelial cells lining the peritoneal cavity. Matrix metalloproteinases (MMP)‐2 and MMP9 have been widely acknowledged to play a role in the neovascularization, attachment, and invasive properties of the endometrium (Santanam et al. 2013).

A flavonoid named 3,6‐dihydroxyflavone (3,6‐DHF) is abundantly present in plant foods including fruits and vegetables (Balasubramanian et al. 2019). As a well‐known chemopreventive drug and potent JNK kinase inhibitor that may effectively shut down TLR2‐mediated (Toll‐like receptor2) signaling pathways, 3,6‐DHF has been previously used to treat a variety of malignancies, including breast cancer. Breast cancer cell migration, invasion, and tumor‐initiating potential are all decreased by 3,6‐DHF (Chen et al. 2016). Yu and Zhou (2018) conducted an in vivo investigation to look into the inhibitory effects of 3,6‐DHF on endometrial stromal cell changes. In this research project, the in vitro model used primary cultivated ovarian ectopic endometrial stromal cells (OvESCs). Participants who had endometriosis gave fresh samples. Forty Sprague Dawley (SD) rats and 21 SCID (severe combined immunodeficient) mice were recruited. For 24 h, OvESCs were exposed to 3,6‐DHF at various concentrations (0–20 M). The findings demonstrated that 3,6‐DHF prevented ectopic endometrial stromal cells from migrating or invading. The endometriosis model group, who got 3,6‐DHF, had smaller lesions. Furthermore, 3,6‐DHF prevents the NICD‐CSL‐MAML complex from attaching to OvESCs, which prevents the production of proteins involved in the Notch signaling pathway in vitro and has an anti‐invasive function. In the 3,6‐DHF group, MMP9 protein expression was markedly and dose‐dependently downregulated, as also indicated in the study of Kapoor et al. (2019). Additionally, 3,6‐DHF elevated E‐cadherin while lowering the mRNA levels of Twist, Snail, and Slug.

An in vitro study on patient‐derived immortalized human ovarian endometriotic stromal cells demonstrated that baicalein suppresses cell proliferation and normal cell cycle progression through the inhibition of cyclins and *cyclin*‐dependent kinases (CDKs) (Park et al. 2024).

Epigallocatechin gallate is one of the main flavonoids. Due to its antioxidant, antiproliferative, and antiangiogenic properties, it results in the initiation of apoptosis and cell cycle arrest (Markowska et al. 2023).

Another study showed the effect of flavonoid extract from *Phaleria macrocarpa* to proliferating factors (MMP‐1, MMP‐3, MMP‐7) in Endometriosis Mice Model (Irwanto, Wiyono, and Wardani 2023).

In another study, the effectiveness of flavonoids on E‐cadherin was also noted. The goal of Hsu et al. (2020) study was to investigate both the in vivo and in vitro effects of ISL on endometriosis. ISL has previously demonstrated anti‐inflammatory, anti‐tumor, anti‐antioxidant, and anti‐proliferation properties (Demirel et al. 2014). Additionally, it has been shown to prohibit cancerous cells from proliferating and migrating and to trigger apoptosis (Sezik et al. 2001). In this research (Skočibušić et al. 2004), they employed End1/E6E7 endometriosis cells and mature (7‐week‐old) female mice. Four groups of mice (*n* = six each) were subjected to subcutaneous injections of estradiol (10 mg/kg) twice weekly. One group received oral administration of a low dose of ISL (1 mg/kg), while another group received a high dose of ISL (5 mg/kg). The control group was administered ISL via gavage. Quercetin was selected as the active control. ISL effectively prevented EMT in endometriotic animals by upregulating the expression of E‐cadherin and downregulating the expression of N‐cadherin, Snail, and Slug in endometriotic lesions. Furthermore, ISL inhibited the growth of End1/E6E7 cells and prevented EMT induced by β‐estradiol. The anti‐endometriotic effects of ISL were achieved through various mechanisms, including inhibition of PCNA expression (along with Bax, Bcl‐2, and caspase‐3), reduction of the anti‐apoptotic protein Bcl‐2 and estrogen receptor expression, and augmentation of Bax and cleaved caspase‐3 expression in endometriotic lesions. These findings demonstrate that ISL retards the development of endometriotic lesions in mice.

**FIGURE 2:**
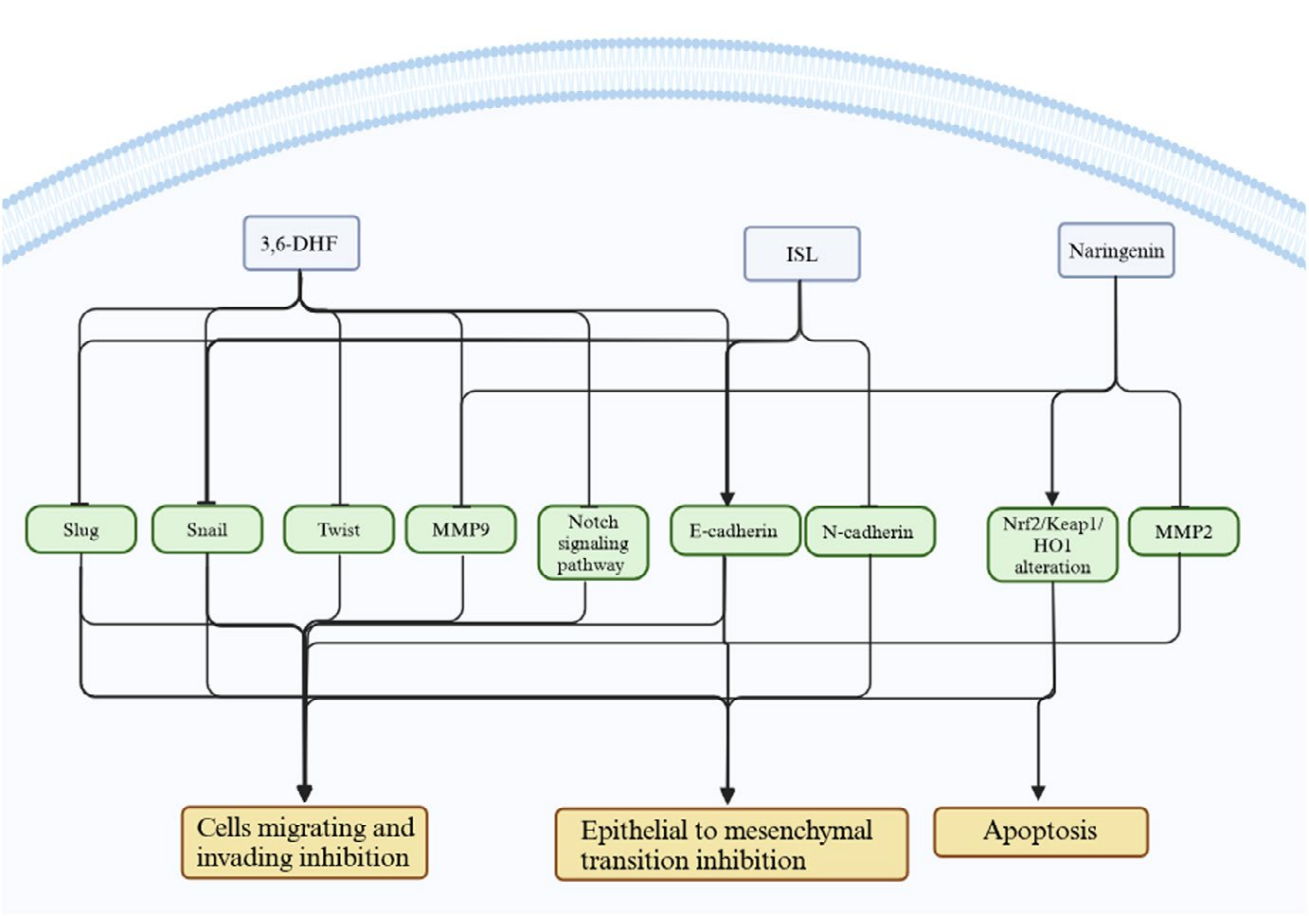
3,6‐DHF, ISL, and naringenin modulate different mediators related to the endometrial cells. The effect of these flavonoids on the mediators leads to apoptosis, inhibition of cell migration and invasion, and epithelial to mesenchymal transition in endometriosis cells. Created with BioRender.com.

In chronic and metabolic illnesses, naringenin, a flavonoid produced from plants, has anti‐proliferative, anti‐inflammatory, and anti‐angiogenic effects (Güner et al. 2000). Kapoor et al. (2019) conducted an in vivo investigation into the course of endometriosis in rats by altering the Nrf2/Keap1/HO1 axis and triggering apoptosis, which is alleviated by naringenin. Adult female SD rats (weighing 180–200 g) were separated into five groups (*n* = 6) for this in vivo study. Endometriotic mice were administered orally with naringenin (50 mg/kg bwt/day) at the time endometriosis was induced in one group and for 21 days in another group. One group was a sham control, and the other was made up of endometriosis control rats. Endometriotic rats were administered orally in the fifth group for 21 days at a low dose of naringenin (0.3 mg/kg bwt/day). Treatment of endometriotic cells with naringenin resulted in a substantial decline in the number of cells migrating to the transwell migration chamber, demonstrating anti‐invasive capabilities. This study found that naringenin decreased the size, number, and proliferation of glands, as well as the volume and weight of endometrial lesions. Moreover, it demonstrated anti‐inflammatory effects by lowering endometriotic lesions' serum TNF‐α and NO levels. Naringenin was used in various quantities for the vitro experiment (0.1–10 M). Naringenin exhibited anti‐proliferative properties, inducing apoptosis and causing mitochondrial membrane injury, resulting in the generation of reactive oxygen species (ROS). The expression levels of Bcl‐2, caspase‐3, Cyt‐c, PCNA, HO1, NQO1, and Keap1 were also restored. Furthermore, naringenin significantly reduced the expressions of TAK1, PAK1, VEGF, and Nrf2. The anti‐metastatic effects of naringenin were evident in the reduced migration of cells and decreased expression of MMP‐2 and MMP‐9. The effect of flavonoids on proliferation and invasion stress is shown in Figure 2.

## 7 Flavonoid–Receptor Interactions and Their Effects on Endometriosis

### 7.1 PPARγ and PPARα Interactions With Flavonoids in Endometriosis

**FIGURE 3:**
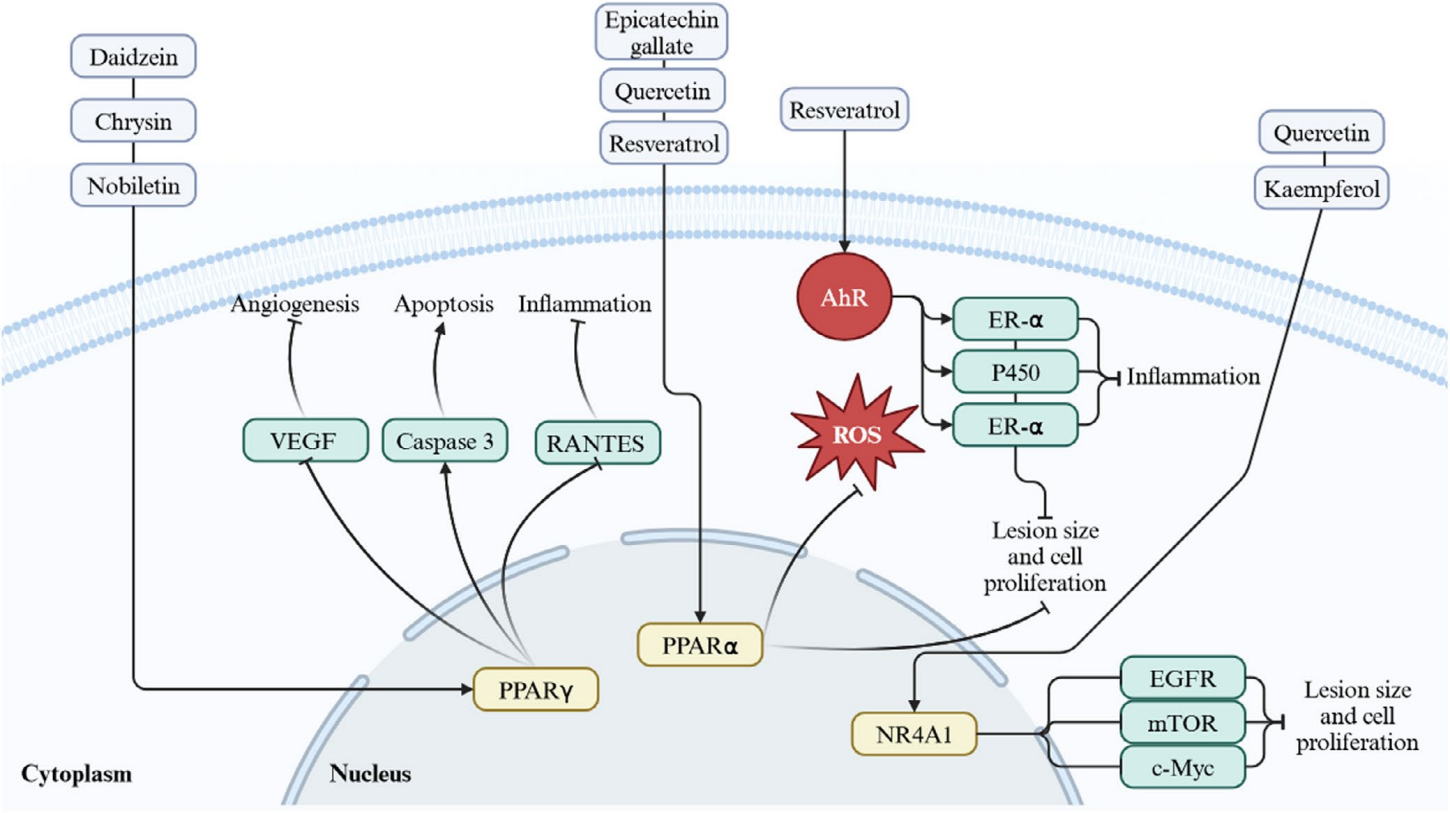
Flavonoid interactions with receptors PPARγ, PPARα, AhR, and NR4A1 in regulating endometriosis‐related pathways, including inflammation, apoptosis, angiogenesis, and lesion proliferation. Created with BioRender.com.

PPARγ plays a pivotal role in the pathogenesis of endometriosis by modulating key biologic pathways, such as inflammation, angiogenesis, and apoptosis. Various studies have investigated its impact, especially in relation to therapeutic approaches. Angiogenesis is critical for the growth of endometriotic lesions, and PPARγ has been shown to inhibit this process. Treatment with rosiglitazone, a PPARγ agonist, in a rat model of endometriosis significantly reduced VEGF levels, thus decreasing blood vessel formation in endometriotic tissues. This led to reduced lesion size and enhanced apoptosis in endometrial cells (Zhang et al. 2021). PPARγ activation also promotes apoptosis in endometriotic tissues. In the study using rosiglitazone, morphological changes consistent with apoptosis, such as vacuolization, were observed in treated tissues, along with increased caspase‐3 expression (Zhang et al. 2021). Pioglitazone, another PPARγ agonist, was shown to reduce the levels of RANTES, a chemokine associated with inflammation, in patients with endometriosis. This reduction in inflammation was associated with improved implantation rates in patients undergoing in vitro fertilization (IVF), suggesting that PPARγ activation could help create a more favorable reproductive environment for those with endometriosis. Flavonoids, such as nobiletin, chrysin, and daidzein, have been shown to regulate PPARγ through various mechanisms, which could be relevant in managing metabolic and inflammatory conditions. Nobiletin, found in citrus fruits, reduces inflammation by activating PPARγ and inhibiting the release of cytokines like TNF‐α and IL‐6, with its effects reversed by PPARγ inhibition (Yue et al. 2024). Chrysin, particularly its derivative YGT‐31, selectively inhibits PPARγ‐Ser273 phosphorylation, mitigating inflammation and enhancing insulin sensitivity without the side effects associated with full agonists (Ma et al. 2024). Daidzein, an isoflavone, acts as a dual agonist for PPARα and PPARγ (Goleij et al. 2024), improving endothelial function and reversing high glucose‐induced damage in cells (Yang et al. 2024). Additionally, research has demonstrated that flavonoids like quercetin and epicatechingallate, through molecular docking, bind effectively to proliferator‐activated receptor gamma (PPARα), a related receptor, enhancing lipid metabolism and potentially reducing disease complications linked to metabolic syndrome (Hassan et al. 2023). Transcriptional studies in endometriosis model rats treated with resveratrol have shown that PPARγ activation is pivotal in mediating the anti‐inflammatory and metabolic regulatory effects of the flavonoid. Resveratrol's influence on lipid metabolism and the insulin resistance pathway underscores its capacity to modulate cellular environments conducive to endometriosis development. By improving glucose tolerance and reducing inflammatory polarization in macrophages, resveratrol facilitates a more balanced immune response, suggesting its therapeutic potential extends beyond simple symptom relief (Wang et al. 2021). Flavonoids have shown potential as effective modulators of PPARα. In a study focused on bovine PPARα, molecular docking analyses of 1000 flavonoids identified two compounds—quercetin‐3‐o‐rhamnoside and (−)‐epicatechin gallate—that exhibited high binding affinity for PPARα, surpassing even synthetic PPARα agonists (Hassan et al. 2023). Chrysin, a dihydroxyflavone, has demonstrated protective effects in animal models of endometrial hyperplasia, where it counteracts estradiol‐induced endometrial thickening by activating PPARα. This activation reduces OS markers like MDA while enhancing the levels of protective antioxidant enzymes such as SOD and GPx. Moreover, it suppresses inflammatory mediators like NFκB and TNF‐α, which are known to perpetuate endometrial lesions in endometriosis (Eid 2022). Similarly, resveratrol, another flavonoid, has been extensively studied for its role in regulating PPARα and PPARγ activity in endometriosis models. In both human ectopic endometrial stromal cells and animal models, resveratrol significantly reduces lesion size and attenuates abnormal lipid metabolism, a hallmark of endometriosis. This effect is partly due to its activation of PPARα, which restores balance in lipid and inflammatory signaling pathways, reducing proliferation and invasiveness of endometriotic cells while promoting apoptosis (Figure 3).

### 7.2 Aryl Hydrocarbon Receptor Interaction With Flavonoids in Endometriosis

Flavonoids have emerged as significant modulators of the aryl hydrocarbon receptor (AhR), a transcription factor involved in processes like detoxification, immune regulation, and cancer. Their role as AhR ligands—either as agonists or antagonists—depends largely on their structure and the cellular context. Some flavonoids, such as unsubstituted flavones, act as AhR agonists, promoting the expression of genes like CYP1A1, while others, particularly hydroxylated flavones, exhibit antagonistic effects (Park et al. 2022). This complex interaction is further highlighted by studies on chalcones, which show that certain hydroxylated forms activate AhR in colon cancer cells, impacting gene expression related to detoxification (Park et al. 2021). However, the effects of flavonoids are highly variable, with some compounds, such as acacetin, acting as strong AhR agonists, while others, like genistein, remain inactive (Park et al. 2019). Flavonoids' dual roles as AhR modulators offer potential therapeutic benefits, particularly in cancer and immune‐related conditions, but their inconsistent effects call for further research. The interplay between flavonoid structure and AhR activity underscores the need for more detailed studies to harness their full pharmacological potential (Yang et al. 2019). In a study conducted by Amaya and colleagues, the impact of resveratrol on the expression of estrogen receptor α (ER‐α), Ki‐67 (a marker for cell proliferation), AhR, and cytochrome P450 enzymes was examined. Their findings revealed that mice administered a combination of estradiol (E2) and progesterone, or E2 with a high dose of resveratrol (60 mg), experienced a decrease in ER‐α and Ki‐67 levels within eutopic endometrial epithelial cells (Amaya et al. 2014). Resveratrol may influence aryl hydrocarbon receptors (AhRs) in endometriosis by inhibiting cholesterol biosynthesis and modulating HMGCR (3‐hydroxy‐3‐methylglutaryl‐coenzyme A reductase) expression and activity. Since AhRs are involved in regulating inflammation and cell proliferation, the antiproliferative and anti‐inflammatory properties of resveratrol could potentially affect AhR signaling pathways, thereby contributing to the suppression of endometriotic lesion growth. This interaction may enhance the therapeutic potential of resveratrol in managing endometriosis (Villanueva et al. 2013; Figure 3).

### 7.3 NR4A1 Interaction With Flavonoids in Endometriosis

Flavonoids exhibit significant modulatory effects on orphan nuclear receptor 4A1 (NR4A1), an important factor in cancer biology. Studies have demonstrated that both quercetin and kaempferol directly bind to NR4A1 with notable affinities, influencing its role in regulating oncogenic pathways. In rhabdomyosarcoma (RMS) cells, these flavonoids inhibit NR4A1‐dependent transactivation, which is critical for cell survival, growth, and invasion. By suppressing pro‐oncogenic genes like PAX3‐FOXO1 and G9a, and interfering with the mTOR signaling pathway, they reduce tumor progression (Shrestha et al. 2021). In vivo studies on animal models further confirm their potential, where kaempferol and quercetin were able to inhibit tumor growth in RMS‐bearing xenografts. The therapeutic efficacy of these compounds is enhanced by their capacity to act as NR4A1 antagonists, making them promising candidates for precision oncology, particularly in cancers overexpressing NR4A1. Further analysis reveals that the interaction of flavonoids with NR4A1 is highly dependent on their hydroxylation patterns, highlighting their selective modulatory effects. This specificity opens avenues for using hydroxyflavones as nutraceutical interventions targeting NR4A1, with the potential to improve the efficacy of existing cancer treatments (Lee et al. 2023). Flavonoids like kaempferol and quercetin have shown potential in targeting the nuclear receptor NR4A1, which is overexpressed in endometriotic tissue. By acting as natural antagonists, they suppress NR4A1 activity, leading to reduced proliferation of endometriotic and cancerous cells through the inhibition of pathways like EGFR, c‐Myc, survivin, and mTOR. Additionally, these compounds influence fibrosis and OS, adding to their therapeutic effects. Animal studies confirm that kaempferol and quercetin reduce the size of endometriotic lesions without affecting body weight, suggesting their promise as nutritional treatments for endometriosis (Zhang et al. 2023; Figure 3).

## 8 Discussion, Conclusions, and Perspectives

Endometriosis is a common gynecologic illness that can cause pain, discomfort, and many other morbidities. These morbidities can affect a person's quality of life (Davoodi et al. 2022). The exact treatment for this disease is still unknown, and only preservative therapies have been investigated for it (Hoyle and Puckett 2022). There are multiple pathways implicated in the development of endometriosis, making it challenging to identify a precise therapeutic strategy. While endometriosis itself is not a malignant condition, certain characteristics such as OS, migration and invasion, angiogenesis, and inflammation, exhibit similarities to tumor cells. This review focuses on the tumor‐like properties of endometriosis targeted by flavonoids, although it acknowledges that these mechanisms are not the exclusive pathological manifestations of the condition. However, they represent crucial complications worthy of attention. The study findings indicate that H2DCF‐DA, naringenin, apigenin, myricetin, DMF, chrysin, and 6,8‐diprenylorobol possess properties that induce OS. While OS is a leading cause of malignancy, it appears to be beneficial in preventing endometriosis. Flavonoids can target molecular pathways associated with OS, including ROS accumulation, lipid peroxidation, GRP78, and eIF2α. By targeting these pathways, flavonoids can enhance OS, leading to the upregulation of apoptosis and cell death in endometriosis. Xanthohumol, isoliquiritigenin, and luteolin were observed to affect angiogenesis. These flavonoids act as anti‐angiogenic agents through two distinct mechanisms: direct targeting of VEGF and indirect targeting of VEGF by inducing macrophages that secrete VEGF, thus promoting angiogenesis in endometriosis. Apigenin, isoliquiritigenin, and luteolin were found to possess anti‐inflammatory properties. These flavonoids exert their effects by targeting NF‐kB, TNF‐α, IL‐1, IL‐6, and endometriosis‐associated macrophages, thereby impeding the progression of endometriosis. Furthermore, 3,6‐dihydroxyflavone, isoliquiritigenin, and naringenin were identified as flavonoids with anti‐invasive activities. These flavonoids affect various molecular mechanisms, including JNK kinase inhibitor, Toll‐like receptor 2, E‐cadherin, apoptosis, N‐cadherin, Snail, Slug, EMT, PCNA expression, Bcl‐2, estrogen receptor, Bax, cleaved caspase‐3, and Nrf2/Keap1/HO1, which collectively contribute to the prevention of endometriosis invasiveness. Flavonoids not only target individual causes of endometriosis but can also affect multiple causes simultaneously. For instance, naringenin affects OS and invasiveness, luteolin impacts inflammation and angiogenesis, apigenin targets inflammation and OS, and isoliquiritigenin influences angiogenesis, inflammation, and invasiveness. Therefore, researchers may consider employing different flavonoids in combination to achieve improved clinical outcomes.

Flavonoid–receptor interactions, particularly with PPARγ, PPARα, AhR, and NR4A1, present compelling therapeutic potential for managing endometriosis. Through PPARγ activation, flavonoids such as nobiletin, chrysin, and daidzein demonstrate anti‐inflammatory and anti‐angiogenic effects, promoting apoptosis in endometriotic tissues and suppressing key inflammatory mediators like TNF‐α and IL‐6. Resveratrol's impact on both PPARγ and PPARα suggests a dual role in modulating lipid metabolism and reducing inflammation, further supporting its use in mitigating the metabolic dysregulation seen in endometriosis. Flavonoids also influence AhR, a receptor critical in immune regulation and detoxification. Resveratrol, by modulating AhR pathways, inhibits cell proliferation and reduces cholesterol biosynthesis, thereby contributing to the suppression of lesion growth. This anti‐proliferative and anti‐inflammatory action positions flavonoid as promising agents for immune modulation in endometriosis. Additionally, the antagonistic effects of flavonoids like quercetin and kaempferol on NR4A1 offer a targeted approach to controlling cell proliferation, OS, and fibrosis in endometriotic tissue. Their ability to inhibit pathways such as EGFR and mTOR underscores their broader role in reducing lesion size and disease progression.

While these findings shed light on the potential preventive and therapeutic effects of flavonoids on endometriosis, the lack of clinical data to support their effects in humans is noteworthy. Additionally, it is essential to assess the safety of these phytochemicals when designing drugs (Goleij et al. 2024). Investigations into the pharmacokinetic and pharmacodynamic properties of these compounds are also necessary and unavoidable.

## Author Contributions

**Pouya Goleij:** investigation (equal), supervision (equal), writing – original draft (equal), writing – review and editing (equal). **Mohanna Khandan:** data curation (equal), formal analysis (equal), investigation (equal), writing – original draft (equal). **Mohammad Amin Khazeei Tabari:** formal analysis (equal), investigation (equal), writing – original draft (equal). **Pantea Majma Sanaye:** formal analysis (equal), investigation (equal), visualization (equal), writing – original draft (equal). **Dorsa Alijanzadeh:** formal analysis (equal), investigation (equal), methodology (equal), writing – original draft (equal). **Afsaneh Soltani:** formal analysis (equal), investigation (equal), writing – original draft (equal). **Zahra Hosseini:** formal analysis (equal), investigation (equal), writing – original draft (equal). **Danaé S. Larsen:** formal analysis (equal), investigation (equal), validation (equal), writing – original draft (equal). **Haroon Khan:** formal analysis (equal), investigation (equal), writing – review and editing (equal). **Alan Prem Kumar:** formal analysis (equal), investigation (equal), writing – review and editing (equal). **Maria Daglia:** conceptualization (equal), supervision (equal), writing – review and editing (equal).

## Conflicts of Interest

The authors declare no conflicts of interest.

## Data Availability

No datasets were generated or analyzed in this study.
